# Glutamate dehydrogenase as a biomarker for mitotoxicity; insights from furosemide hepatotoxicity in the mouse

**DOI:** 10.1371/journal.pone.0240562

**Published:** 2020-10-09

**Authors:** Rachel J. Church, Shelli J. Schomaker, J. Scott Eaddy, Germaine G. Boucher, John M. Kreeger, Jiri Aubrecht, Paul B. Watkins

**Affiliations:** 1 Institute for Drug Safety Sciences, University of North Carolina at Chapel Hill, Chapel Hill, North Carolina, United States of America; 2 Division of Pharmacotherapy and Experimental Therapeutics, Eshelman School of Pharmacy, University of North Carolina at Chapel Hill, Chapel Hill, North Carolina, United States of America; 3 Pfizer Inc., Groton, Connecticut, United States of America; University of Navarra School of Medicine and Center for Applied Medical Research (CIMA), SPAIN

## Abstract

Glutamate dehydrogenase (GLDH) is a liver-specific biomarker of hepatocellular damage currently undergoing qualification as a drug development tool. Since GLDH is located within the mitochondrial matrix, it has been hypothesized that it might also be useful in assessing mitotoxicity as an initiating event during drug-induced liver injury. According to this hypothesis, hepatocyte death that does not involve primary mitochondrial injury would result in release of intact mitochondria into circulation that could be removed by high speed centrifugation and result in lower GLDH activity measured in spun serum vs un-spun serum. A single prior study in mice has provided some support for this hypothesis. We sought to repeat and extend the findings of this study. Accordingly, mice were treated with the known mitochondrial toxicant, acetaminophen (APAP), or with furosemide (FS), a toxicant believed to cause hepatocyte death through mechanisms not involving mitotoxicity as initiating event. We measured GLDH levels in fresh plasma before and after high speed centrifugation to remove intact mitochondria. We found that both APAP and FS treatments caused substantial hepatocellular necrosis that correlated with plasma alanine aminotransferase (ALT) and GLDH elevations. The plasma GLDH activity in both the APAP- and FS- treated mice was not affected by high-speed centrifugation. Interestingly, the ratio of GLDH:ALT was 5-fold lower during FS compared to APAP hepatotoxicity. Electron microscopy confirmed that both APAP- and FS-treatments had resulted in mitochondrial injury. Mitochondria within vesicles were only observed in the FS-treated mice raising the possibility that mitophagy might account for reduced release of GLDH in the FS-treated mice. Although our results show that plasma GLDH is not clinically useful for evaluating mitotoxicity, the GLDH:ALT ratio as a measure of mitophagy needs to be further studied.

## Introduction

Drug-induced liver injury (DILI) is a serious concern for pharmaceutical companies and clinicians alike, representing the number one cause of acute liver failure in the United States [1]. Serum alanine aminotransferase (ALT) is considered the gold standard biomarker for detection and monitoring of hepatotoxicity; however, elevations in ALT are not specific to liver injury. Since ALT is also present in myocytes, extreme exercise, muscle disease, and seizures have all been reported to result in release of ALT into circulation [2, 3].

Glutamate dehydrogenase (GLDH) is a large enzyme that is embedded within the mitochondrial matrix. Unlike cristae-rich mitochondria that are abundant in muscle tissue, matrix-rich mitochondria are highly concentrated in the liver and studies have demonstrated that GLDH activity outside of the liver is minimal [4]. As opposed to ALT, GLDH is not elevated in patients with muscle disorders [5, 6]. Therefore, GLDH might be particularly useful for diagnosing liver injury in patients with underlying muscle impairments [6]. Additionally, because GLDH has a shorter half-life in circulation compared to ALT in humans, this biomarker may give a more accurate reflection of the concurrent liver injury [7–9]. Research in rodents and humans has indicated that basal levels of GLDH in blood are low and become substantially increased in response to liver injury [9–14]. GLDH has also shown value as a prognostic biomarker of outcome in the clinic following acetaminophen (APAP) overdose [13]. Based on these and other observations, a Qualification Plan for GLDH was accepted by the FDA in 2020, encouraging submission of a Full Qualification Package for GLDH use as a biomarker of liver injury in patients with underlying muscle injury [15].

In addition to representing a general biomarker of hepatotoxicity, GLDH has also been proposed as a potential mechanistic biomarker [16]. The idea is that if liver cell death occurs due to mitotoxicity, GLDH should leak from damaged mitochondria into hepatocyte cytosol prior to liver cell death and few intact mitochondria would end up in circulation. However, if hepatocyte death occurred by mechanisms not involving mitotoxicity, intact mitochondria could be released during hepatocyte death. It was proposed that the role of mitochondria in hepatotoxicity might only be inferred if intact mitochondria were removed during sample processing. The plausibility of this hypothesis is supported by the recent observation that whole and functional mitochondria can be found in circulation [17]. However, to date the only support for use of GLDH as a biomarker of mitotoxicity is a single study performed in mice treated with a toxic dose of APAP, a toxicant known to cause mitotoxicity, and mice treated with a toxic dose of furosemide (FS), a toxicant not believed to cause mitotoxicity [10]. In this study, all fresh blood was subjected to high speed centrifugation that would remove intact mitochondria. A similar extent of liver injury was produced by both treatments based on histopathology and peak elevations in serum ALT. In the APAP-treated mice, GLDH levels were substantially elevated but GLDH levels were not significantly elevated in the FS-treated mice. The authors assumed that this was because intact mitochondria had been released during liver injury due to FS but not (or to a much lower extent) in the APAP-treated animals. However, in this study, GLDH was not measured prior to the highspeed centrifugation step to test this hypothesis more directly.

Because the ability to use GLDH to inform mechanisms of liver injury would be an important advance, we sought to repeat this study but also to measure GLDH prior to the high-speed spin.

## Materials and methods

### Experimental animals

Male C57BL6/J mice (approximately eight weeks of age) were purchased from the Jackson Laboratory (Bar Harbor, ME) and were housed in polycarbonate cages on a 12-h light-dark cycle. Mice were provided standard diet *ad libitum*. Half the mice were fasted overnight (approximately 17–18 hours) prior to dosing and food was provided to all mice immediately following dosing. Reverse osmosis water was available to all mice, *ad libitum* throughout the entire course of the study. All animal use was conducted under protocols approved by local Institutional Animal Care and Use Committee (IACUC) boards.

### Animal studies

Because our aim was to reproduce the work conducted by McGill *et al*. [10], male C57BL6/J mice were selected for use in our study. The number of mice per treatment was selected to minimize sample size while providing sufficient animals to control for variance within treatment groups. We determined that the optimum volume of plasma, per animal, necessary for the downstream analyses we planned was approximately 1mL. However, the total expected plasma volume, per mouse, was closer to half of this required quantity. Therefore, we determined it may have been necessary to pool samples for one or more of the downstream analyses in this study. Statistical analyses can be completed on n = 3 animals per group. Plasma pools containing sample from two mice each would give volumes sufficient to complete analyte quantifications; therefore, n = 6 mice per treatment group was the minimum number of animals necessary to facilitate the detection of statistically significant differences between groups in quantifications made in pooled samples.

Animal experimentation was conducted as described in S1 Table. Briefly, mice were randomized for dosing of APAP, FS, or vehicle (0.9% saline or PBS pH 8.5–9 for APAP and FS, respectively). Mice receiving APAP or saline were fasted overnight immediately prior to dosing. Mice were given compound or vehicle by intraperitoneal (*i*.*p*.) instillation and food was returned to all animals immediately following dosing. Mice were euthanized under deep isoflurane-induced anesthesia via cardiac puncture followed by thoracotomy 24h after dosing.

### Clinical chemistry

**Fig 1:**
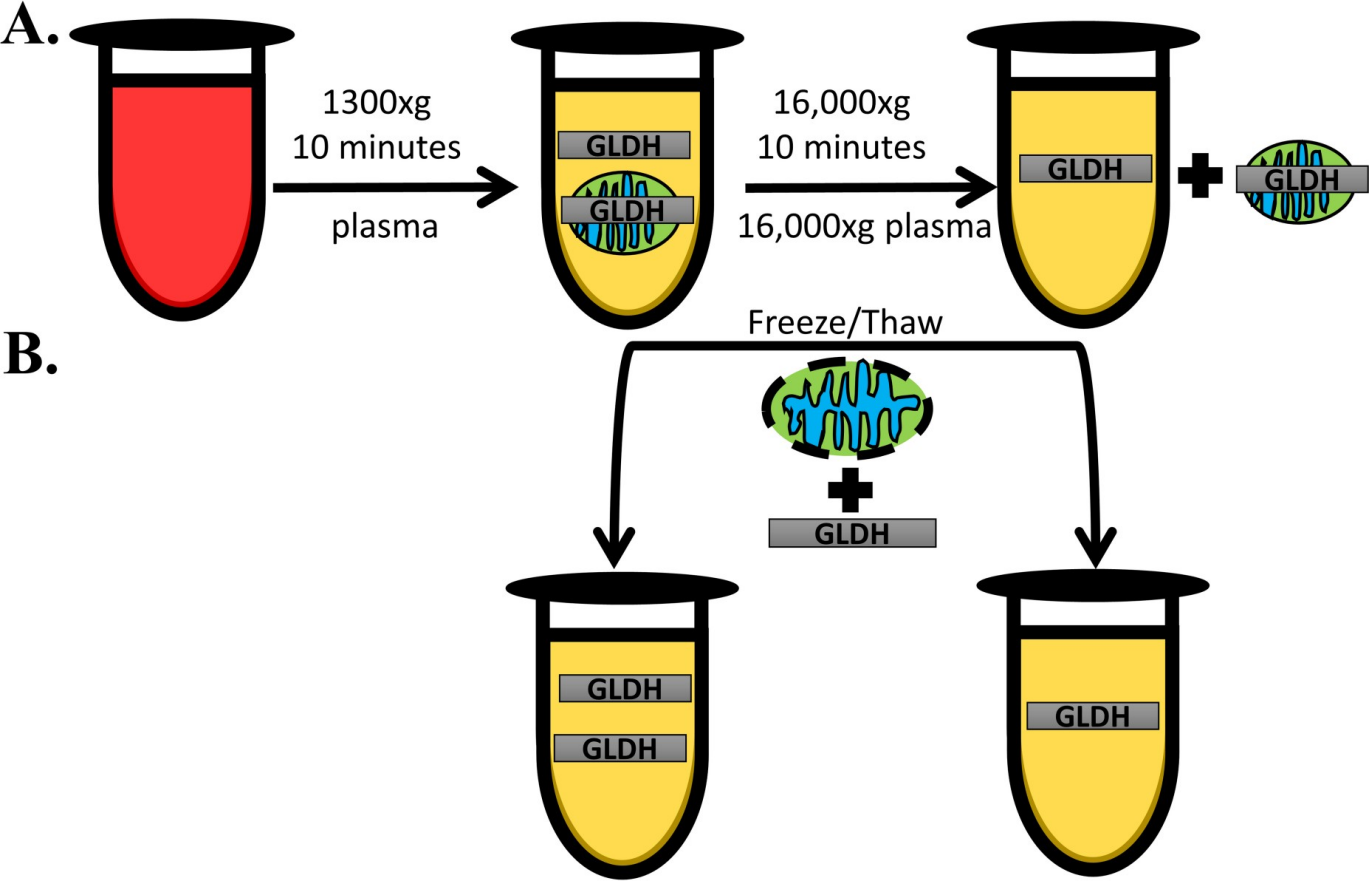
Effect of sample processing on GLDH quantification. GLDH is located within the mitochondrial matrix and it has been hypothesized that failure to remove intact mitochondria during blood processing for serum/plasma can alter GLDH quantifications. Therefore, we processed blood samples collected from mice in multiple ways to determine if mitochondrial removal impacted measurements (**A**). All blood samples initially underwent a standard low speed spin to remove red blood cells and debris and to isolate plasma. An aliquot from each of these samples underwent an additional high-speed spin that was forceful enough to pellet any intact mitochondria that may have been present in the standard plasma to obtain post-mitochondrial supernatant (16,000xg plasma). GLDH was then quantified in the fresh samples with the expectation that if the assay reagents can penetrate the mitochondrial membrane, removal of intact mitocondria would result in a reduction in GLDH levels. However, if the reagents cannot penetrate the undamaged membrane, the presence of intact mitochondria in plasma will not be consequential until these mitochondria are lysed (**B**). We performed three freeze/thaw cycles on our samples which would presumably lyse any mitochondria that remained in the plasma samples. This lysis should liberate GLDH from damaged mitochondria and result in a rise in GLDH levels if the assay reagents cannot detect GLDH in intact mitochondria.

Blood was collected at necropsy from each animal in an EDTA-containing tube and processed for plasma using the standard centrifugation procedure (1300xg for 10 minutes at 4°C; “plasma”) and for mitochondria-depleted plasma using an additional high-speed centrifugation step (16,000xg for an 10 minutes at 4°C; “16,000xg plasma”) as described in Fig 1. Clinical chemistry measurements were made at Pfizer on a Siemens ADVIA 2400 Automated Chemistry System. Measurements of fresh ALT (Siemens), aspartate aminotransferase (AST; Siemens), and GLDH (Randox Labs Ltd, Roche) levels were made immediately upon receipt of samples (~48h following necropsy). Plasma samples were then freeze/thawed three times to lyse any intact mitochondria and then biomarker measurements were made again. Reported group averages were determined from plasma samples that underwent three freeze/thaw cycles. Individual mouse quantifications for plasma ALT, AST, and GLDH are given in **S1 Dataset**.

### Histology

Formalin fixed liver tissue from all rodents was paraffin-embedded and stained with hematoxylin and eosin (H&E). Additional samples were collected from all mice for and fixed in Karnovsky’s fixative for EM. Selected samples (based on outcome of H&E analysis) were processed to block stage and prepared for electron microscopic examination. The samples were post-fixed in 1% osmium tetroxide, dehydrated through a graded alcohol series, transitioned through propylene oxide and embedded in epoxy resin. Blocks were sectioned on Leica ultramicrotome at 70–90 nm, contrast stained with uranyl acetate and lead citrate and evaluated using a Hitachi H-7100 transmission electron microscope. Liver histopathology and EM were analyzed by an ACVP Board Certified Pathologist.

### Statistics

Statistics were performed using GraphPad Prism v. 7.0 (GraphPad Software). Statistical significance between groups for each biomarker was determined using a Student’s t test. Correlation between ALT and GLDH was calculated with Pearson’s r. Data was considered statistically significant if *p*<0.05.

## Results

We sought to reproduce the findings of McGill *et al*. and thus provide support for the utility of GLDH as a mechanistic biomarker for mitochondrial toxicity *in vivo* [10]. Thus, as done by McGill *et al*., we compared GLDH release following hepatotoxicity induced by APAP, a model hepatotoxicant that causes primary mitochondrial dysfunction with hepatotoxicity induced by FS, which is believed to occur without inducing primary mitochondrial injury [10]. We assessed GLDH levels in fresh plasma subjected to a high-speed centrifugation to pellet intact mitochondria (16,000xg plasma) as was done in the prior study. In addition, we assessed GLDH levels in the fresh plasma prior to the high-speed spin (this was not reported in the prior study [10]). In addition, to exclude the possibility that GLDH contained within intact mitochondria is not measured by the assay, we repeated our assays after three freeze/thaw cycles that should disintegrate mitochondria (Fig 1).

### Acetaminophen-induced liver injury in mice

**Fig 2:**
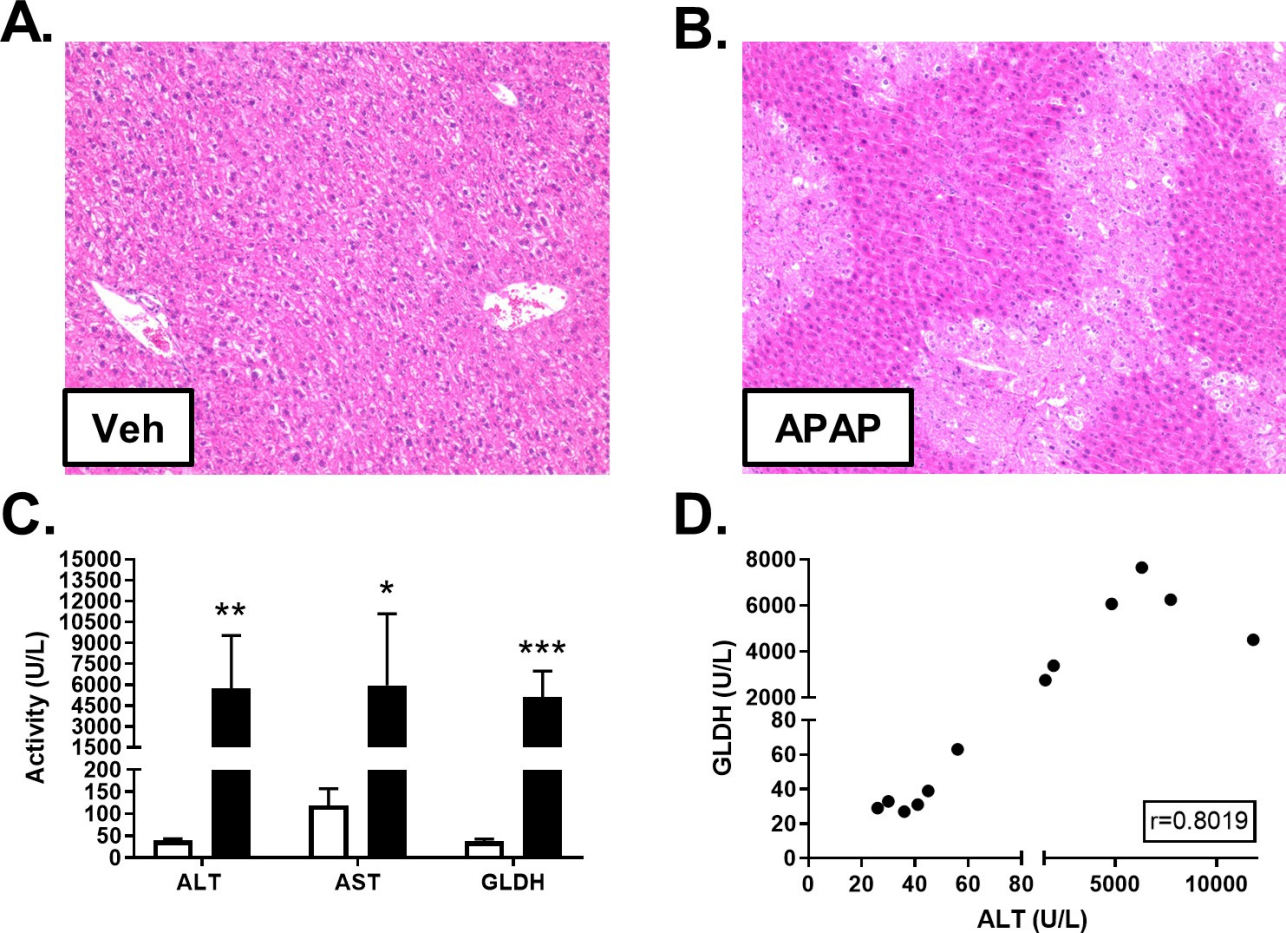
Effect of APAP on liver histology and biomarkers. Male C57BL/6J mice received a single intraperitoneal administration of vehicle (Veh; 0.9% saline) or APAP (300 mg/kg). Liver tissue and blood were collected 24h post-dosing. H&E staining revealed APAP-induced hepatocellular necrosis. Representative photomicrographs at 10X magnification are shown for vehicle- (**A**) and APAP-dosed mice (**B**). ALT, AST and GLDH were quantified in freeze/thawed plasma samples from Veh (white) and APAP-treated (black) mice. Significance was *p<0.05, **p<0.01, or ***p<0.001 (**C**). Pearson’s r was calculated to explore the correlation between ALT and GLDH quantities measured in plasma (**D**).

Male C57BL6/J mice were administered APAP or vehicle, as described in S1 Table. Histopathological analysis revealed that mice dosed with APAP, but not vehicle, displayed centrilobular necrosis 24h post-dosing (Fig 2 and 2B). As expected, plasma ALT and AST were significantly elevated in APAP-dosed animals, compared to controls (Fig 2C and S1 Dataset). APAP-dosed mice also had significantly elevated levels of GLDH in plasma, compared to controls (Fig 2C and S1 Dataset). When all mice were considered, serum ALT and GLDH levels were highly correlated (r = 0.8019; Fig 2D).

### FS-induced liver injury in mice

**Fig 3:**
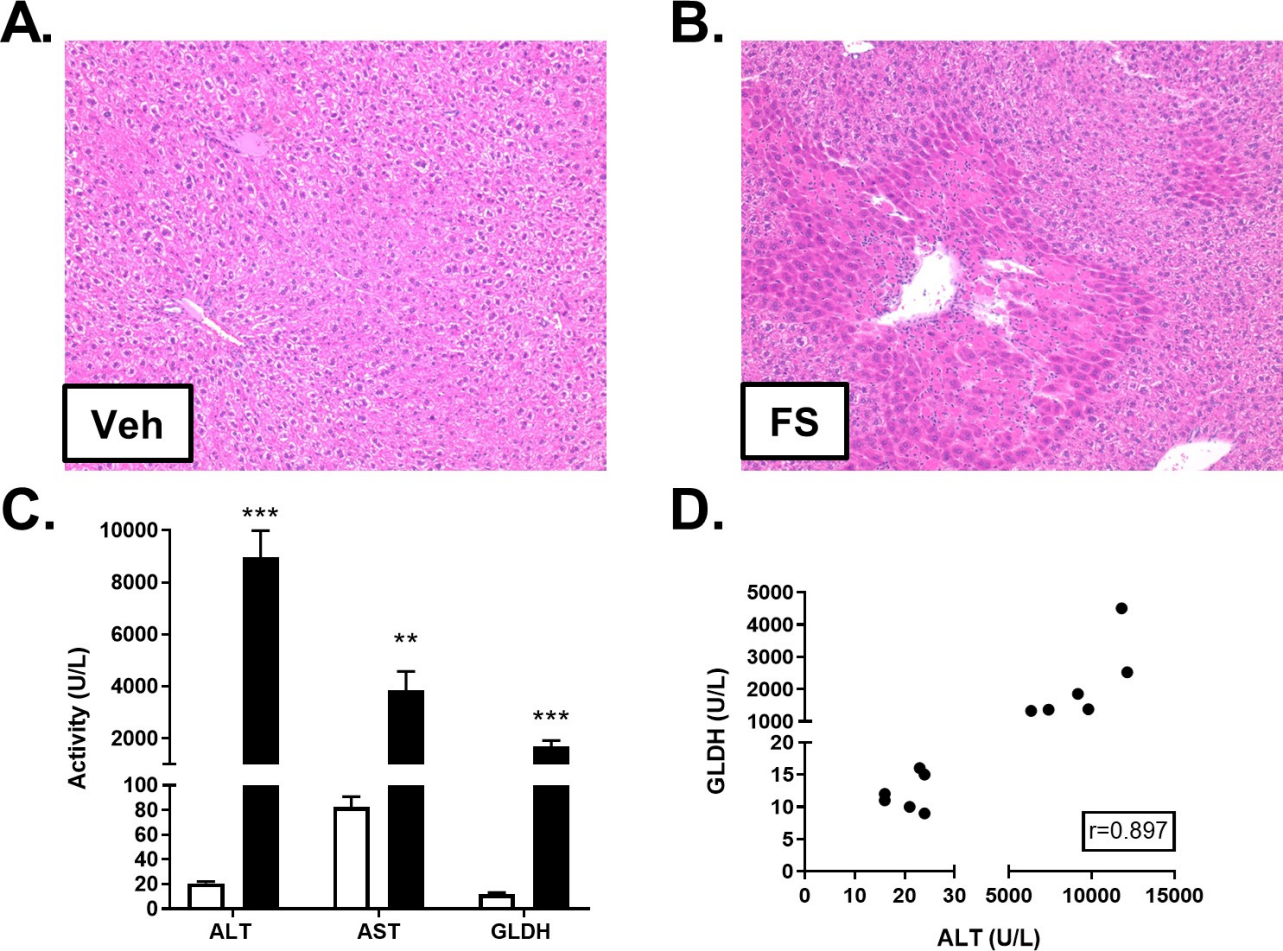
Effect of FS on liver histology and biomarkers. Male C57BL/6J mice received a single intraperitoneal administration of vehicle (Veh) or FS (400 mg/kg). Liver tissue and blood were collected post-dosing. H&E staining revealed FS-induced hepatocellular necrosis and inflammatory cell infiltration. Representative photomicrographs at 10X magnification are shown for vehicle- (**A**) and FS-treated mice (**B**). ALT, AST, and GLDH were quantified in freeze/thawed plasma samples from vehicle (white) and FS-treated (black) mice. Significance was **p<0.01 or ***p<0.001 (**C**). Pearson’s r was calculated to explore the correlation between ALT and GLDH quantities measured in plasma (**D**).

An additional cohort of mice was administered a single 400 mg/kg toxic dose of FS (as described in S1 Table), a compound believed to cause a hepatocellular injury comparable to APAP in mice without being mitotoxic [18]. Twenty-four hours post-dosing, liver histopathology revealed that mice exposed to FS developed centrilobular necrosis, the extent of which appeared to be comparable to that observed in the APAP-dosed mice (Fig 3A and 3B). Additionally, FS-dosed animals had infiltration of inflammatory cells in necrotic areas, a finding that was not observed in APAP-treated mice. As expected, FS administration resulted in significantly elevated plasma levels of ALT and AST, compared to controls (Fig 3C and S1 Dataset). Unexpectedly, FS-dosed animals also displayed large plasma GLDH elevations which correlated with ALT levels (Fig 3C and 3D and S1 Dataset). However, the ratio of mean GLDH to ALT levels was considerably lower during FS toxicity than during APAP toxicity (5.3-fold vs. 1.1-fold lower in FS and APAP toxicities, respectively).

### Effect of plasma processing on GLDH levels in APAP-dosed mice

**Fig 4:**
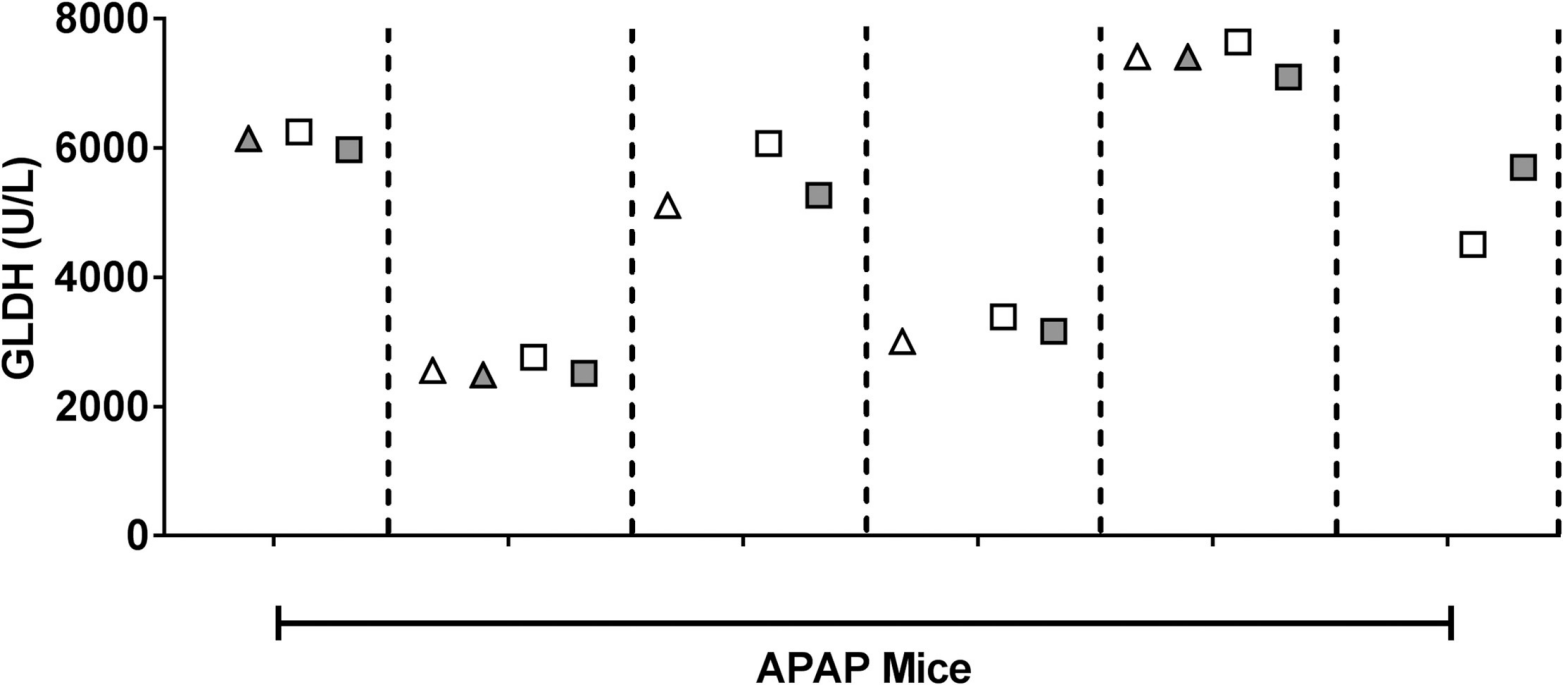
Effect of high-speed spin and freeze/thaw on GLDH levels after APAP administration. Plasma from APAP-dosed mice was isolated with only a standard low-speed spin (white) or with the addition of a high-speed spin (gray). GLDH was quantified in “fresh,” unfrozen samples (triangles) and again in samples that had undergone three freeze/thaw cycles (squares). Some fresh samples froze during transit and could not be measured, resulting in missing symbols. Each tick on the X-axis represents an individual APAP-dosed mouse and each mouse’s samples are separated by a vertical dotted line.

We processed mouse blood for plasma in several ways as described in Fig 1. GLDH was measured in fresh, unfrozen plasma with standard processing and in plasma subjected to an additional 16,000xg spin to remove mitochondria. GLDH was then re-measured in these samples following three freeze/thaw cycles. As shown in Fig 4, performing a high-speed spin on the unfrozen samples collected from the APAP-treated mice did not affect levels of GLDH. In addition, freeze/thawing the samples three times had no effect on the GLDH activity in any of the samples. The results were similar when plasma from vehicle-treated mice underwent the same processing (S1 Dataset).

### Effect of plasma processing on GLDH levels in FS-dosed mice

**Fig 5:**
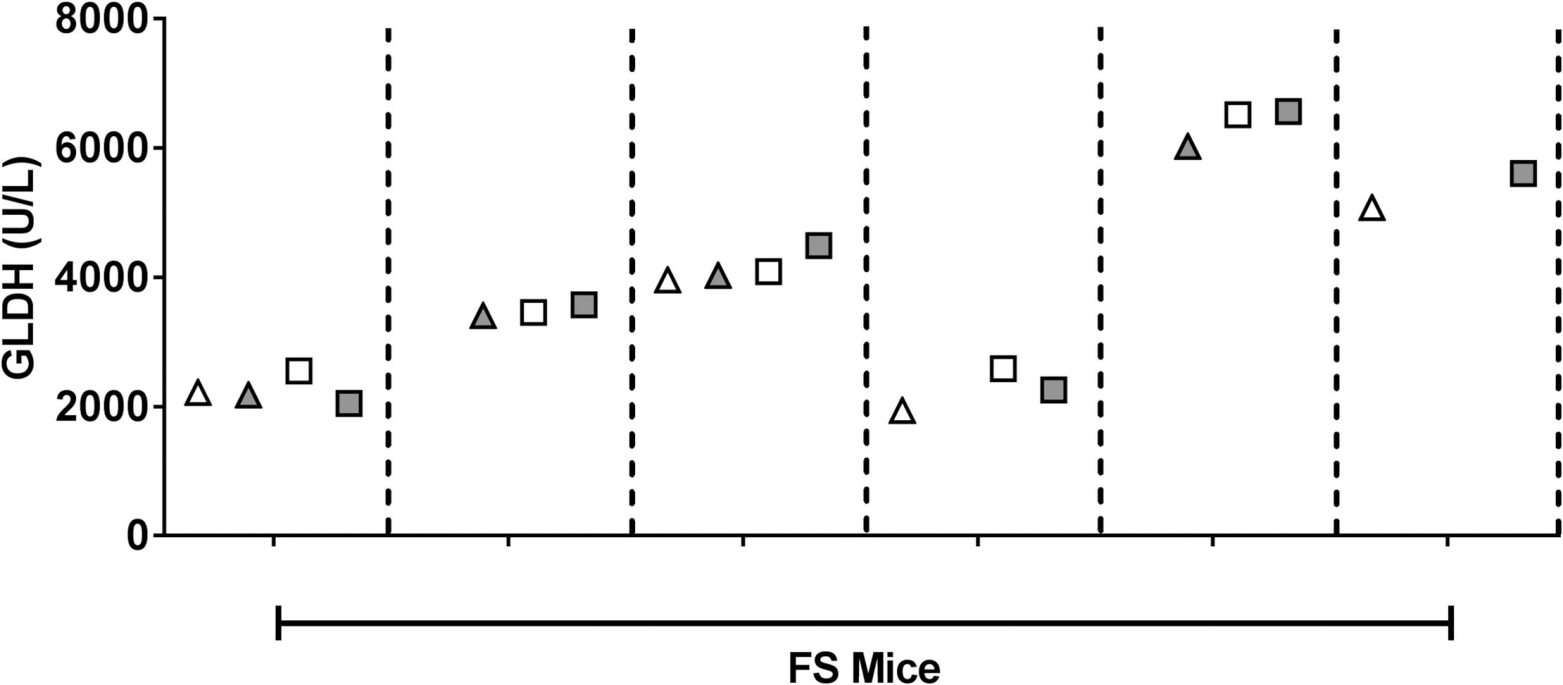
Effect of high-speed spin and freeze/thaw on GLDH levels after FS administration. Plasma from FS-dosed mice was isolated with only a standard low-speed spin (white) or with a subsequent high-speed spin (gray). GLDH was quantified in “fresh,” unfrozen samples (triangles) and again in samples that had undergone three freeze/thaw cycles (squares). Some fresh samples froze during transit and could not be measured, resulting in absent symbols. Each tick on the X-axis represents an individual FS-treated mouse and each mouse’s samples are separated by a vertical dotted line.

Although we identified mitochondrial damage concurrent with GLDH changes in the FS-treated mice (see below), the GLDH to ALT ratio in plasma was 5-fold lower than what we observed in APAP-treated mice. One possibility to account for this ratio difference would be that less mitotoxicity occurred with FS treatment in which case more mitochondria should have been released into circulation in their intact form. However, GLDH levels in fresh plasma were unaffected after the high-speed spin (Fig 5 and S1 Dataset). Subjecting the plasma or 16,000xg plasma to three freeze/thaw cycles to disrupt intact mitochondria also did not affect the GLDH levels.

### Electron microscopy assessment of mitochondrial damage in APAP- and FS-dosed mice

**Fig 6:**
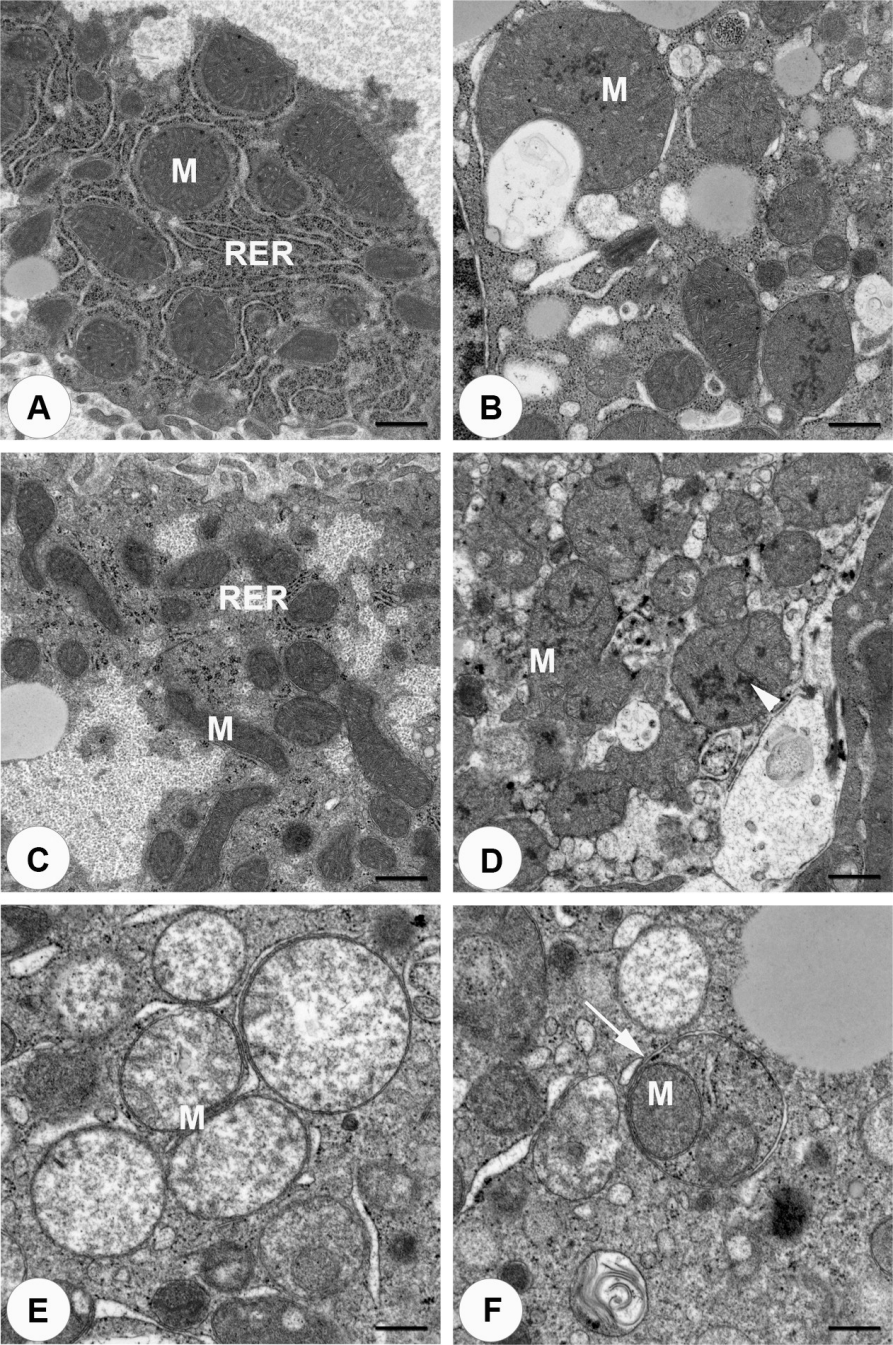
Mitochondrial integrity after compound treatment. The integrity of mitochondria in the liver were assessed by EM and photomicrographs were taken at 10,000X (A-D, bar = 600nm or 15,000X (E,F, bar = 400 nm) magnification. Representative images from APAP-vehicle (A), APAP (B), FS-vehicle (C) and FS (D) dosed mice are shown. Both APAP and FS-dosed animals exhibited megamitochondria and electron dense areas in the mitochondrial matrix (arrow head). FS dosed animals also exhibited swollen mitochondria with dispersion of mitochondrial matrix and blunted cristae (E) and mitochondria within vesicles (F). Abbreviations: M, mitochondria; RER, rough endoplasmic reticulum.

To assess mitochondrial damage, liver sections from vehicle and compound-treated mice underwent EM (Fig 6). At 10,000X magnification, vehicle treated controls had normal appearing mitochondria (Fig 6A and **6C**). As expected, mitochondrial damage was observed following treatment with APAP (Fig 6B). In these animals, megamitochondria were observed along with the presence of electron dense areas in the mitochondrial matrix of some of the mitochondria. Unexpectedly, EM analysis of FS-treated mice also identified substantial injury to mitochondria (Fig 6D). Mitochondria appeared swollen with dispersion of mitochondrial matrix and blunted cristae (Fig 6E). Some mitochondria also contained electron dense areas in the mitochondrial matrix. Interestingly, some mitochondria appeared to be contained within vesicles consistent with mitophagy (Fig 6F).

## Discussion

GLDH is a liver-specific biomarker for hepatocellular injury. It has been proposed that it can also be used to implicate mitotoxicity as an initiating mechanism for drug-induced liver injury [16]. The concept is that hepatocyte death occurring without mitotoxicity will result in release into circulation of intact mitochondria which can be removed from freshly prepared plasma by an additional high-speed spin. There has been only one study that supports this potentially important hypothesis [10], but the necessity for removal of intact mitochondria from blood was assumed and not directly tested.

In agreement with the prior study [10], we found that mice given a toxic dose of APAP developed significantly elevated levels of plasma GLDH. The GLDH values were not affected by the high-speed spin or by three freeze thaw cycles also consistent with the absence of intact mitochondria. These results were expected given that APAP is known to elicit significant mitochondrial damage as a primary mechanism of liver injury [19], and mitochondrial damage by APAP was observed by electron microscopy.

In further agreement with the prior study [10], FS treatment resulted in comparable liver injury to that produced by APAP based on histology and plasma levels of ALT. But in contrast to the prior study, the GLDH levels after high-speed centrifugation were quite high. Moreover, the levels were similar to those obtained prior to the high-speed spin. Likewise, three freeze thaw cycles had no effect on the measured GLDH levels in any of the samples. We conclude that a negligible amount of intact mitochondria were present in the fresh plasma samples, consistent with the fact that mitochondrial injury was produced by FS treatment as judged by EM.

Nonetheless, our results are qualitatively similar to those in the prior study [10] in that the ratio of GLDH to ALT was about 5 fold lower in the FS-treated animals than in the APAP-treated animals. In the prior study [10], GLDH was not measured in plasma prior to the high-speed spin and it was assumed that the low values were due to removal of intact mitochondria. Our data do not support this assumption.

The markedly higher GLDH levels observed in our FS-treated mice compared to the prior study may relate to the greater liver injury in our FS-treated mice. (ALT ~9000 U/L vs. ~2000 U/L, respectively) [10]. Although we attempted to exactly replicate the conditions in the previous study, it appears that our mice were more susceptible to the effects of FS, and therefore had more advanced injury and perhaps more opportunity for secondary mitochondrial damage. It is possible that the infiltration of inflammatory cells observed in the FS- but not in the APAP-treated mouse livers promoted mitochondrial injury after hepatocyte death (and ALT release) had been initiated by other FS-mediated mechanisms. EM examination of the liver at earlier time points during liver injury might therefore have demonstrated intact mitochondria. However, if this were the case, we would expect an initial release of intact mitochondria in the FS-treated mice and, given that intact mitochondria can survive in circulation [17], we would have expected a detectable amount to be present at the 24 hr sacrifice. Therefore, we would have expected fall in GLDH levels following high-speed centrifugation or an increase after freeze thawing, which we did not observe.

Because we sampled blood at only one time point, there is a possibility that differences in time course of injury combined with differences in plasma half-lives of ALT and GLDH could contribute to the changes in ratio we observed. This seems unlikely since the low GLDH:ALT ratio was obtained at a time (24 hours) when the extent of FS injury should have peaked [10]. However, to our knowledge the plasma half-lives of liver enzymes are not known in the mouse and this issue could be further explored with a full time course study. We believe it may be important that some mitochondria in the FS-treated livers were observed within vesicles, consistent with mitophagy which might cause degradation of GLDH prior to release from the cell. Mitophagy may also account for why release of cytochrome 5 into liver cytosol was not observed in a prior study of FS hepatotoxicity in mice [20]. Although previous work demonstrates that APAP hepatotoxicity can induce mitophagy in mice [21], this finding was only observed in the livers of the FS-treated mice in our study, suggesting it may occur more frequently in FS-induced vs. APAP-induced liver injury. The notion is that mitophagy could account for the relative reduction in the GLDH to ALT ratio we observed in FS-treated mice. Furthermore, previously published data also lends credibility to this idea. In a model of liver injury induced by chronic exposure to carbon tetrachloride, rats that received melatonin had a serum GLDH to ALT ratio that was lower than rats that only received carbon tetrachloride [22]. Melatonin was hypothesized to increase mitophagy in this model which was confirmed by EM analysis.

Finally, given the central role of mitochondrial membrane permeabilization in cell death pathways, mitochondrial injury is probably a frequent occurrence during DILI independent of initial events, as is likely the case with FS. This will make it difficult to identify a biomarker that will pinpoint an initiating role for mitochondrial toxicity.

In summary, our data are not consistent with release of intact mitochondria during FS hepatotoxicity. Whereas our data supports GLDH as a biomarker of hepatocellular injury, our data do not support GLDH as a clinically applicable biomarker of mitochondrial damage. Nevertheless, the observed association of decrease of GLDH:ALT ratio with mitophagy deserves further study.

## Supporting information

S1 Table Description of animal experiments. (DOCX)

S1 Dataset Individual mouse clinical chemistry values. (XLSX)
