# Supplementary material for: Glutamate dehydrogenase as a biomarker for mitotoxicity; insights from furosemide hepatotoxicity in the mouse
Source: PLoS One. 2020 Oct 9;15(10):e0240562. doi: 10.1371/journal.pone.0240562 (PMC7546462; doi:10.1371/journal.pone.0240562)
Supplement: S1 Table — (DOCX) [file pone.0240562.s001.docx]

**S1 Table: Description of Animal Experiments**

| **Strain** | **Group Size**  **(n)** | **Compound** | **Dose (mg/kg)** | **Route** | **O/N Pre-Dose Fast** |
| --- | --- | --- | --- | --- | --- |
| C57BL/6J | 6 | 0.9% Saline | -- | *i.p.* | Yes |
| C57BL/6J | 6 | APAP | 300 | *i.p.* | Yes |
| C57BL/6J | 6 | PBS pH 8.5-9.0 | -- | *i.p.* | No |
| C57BL/6J | 6 | FS | 400 | *i.p.* | No |

Abbreviations: O/N, overnight; APAP, acetaminophen; PBS, phosphate buffered saline; FS, furosemide
